# Light-Induced Reactions within Poly(4-vinyl pyridine)/Pyridine Gels: The 1,6-Polyazaacetylene Oligomers Formation

**DOI:** 10.3390/molecules26226925

**Published:** 2021-11-17

**Authors:** Evgenia Vaganova, Dror Eliaz, Ulyana Shimanovich, Gregory Leitus, Emad Aqad, Vladimir Lokshin, Vladimir Khodorkovsky

**Affiliations:** 1 Department of Molecular Chemistry and Materials Science, Weizmann Institute of Science, Rehovot 7610001, Israel; evgenia.vaganov@weizmann.ac.il (E.V.); dror.eliaz@weizmann.ac.il (D.E.); 2 Chemical Research Support Department, Weizmann Institute of Science, Rehovot 7610001, Israel; gregory.leitus@weizmann.ac.il; 3 DuPont Electronics & Industrial, Marlborough, MA 01752, USA; 4 Aix Marseille Univ, CNRS UMR 7325, Centre Interdisciplinaire de Nanoscience de Marseille (CINaM) Campus de Luminy, CEDEX 09, 13288 Marseille, France; vladimir.lokchine@univ-amu.fr

**Keywords:** photochemistry, aromatic heterocycle, pyridine, electroconducting polymer

## Abstract

Cyclic 6-membered aromatic compounds such as benzene and azabenzenes (pyridine, pyridazine, and pyrazine) are known to be light-sensitive, affording, in particular, the Dewar benzene type of intermediates. Pyridine is known to provide the only Dewar pyridine intermediate that undergoes reversible ring-opening. We found that irradiation of photosensitive gels prepared from poly(4-vinyl pyridine) and pyridine at 254 or 312 nm leads to pyridine ring-opening and subsequent formation of 5-amino-2,4-pentadienals. We show that this light-induced process is only partially reversible, and that the photogenerated aminoaldehyde and aminoaldehyde-pending groups undergo self-condensation to produce cross-linked, conjugated oligomers that absorb light in the visible spectrum up to the near-infrared range. Such a sequence of chemical reactions results in the formation of gel with two distinct morphologies: spheres and fiber-like matrices. To gain deeper insight into this process, we prepared poly(4-vinyl pyridine) with low molecular weight (about 2000 g/mol) and monitored the respective changes in absorption, fluorescence, ^1^H-NMR spectra, and electrical conductivity. The conductivity of the polymer gel upon irradiation changes from ionic to electronic, indicative of a conjugated molecular wire behavior. Quantum mechanical calculations confirmed the feasibility of the proposed polycondensation process. This new polyacetylene analog has potential in thermal energy-harvesting and sensor applications.

## 1. Introduction

Aromatic organic compounds are known to undergo a variety of photochemical conversions. Thus, upon UV irradiation, benzene develops yellow coloration owing to the formation of a mixture of unsaturated compounds. The photo-process strongly depends on the wavelength of irradiation and temperature. The isolated products were fulvene, Dewar benzene, and benzvalene, which revert back to benzene and hexadienyne isomers, along with a polymeric material formed irreversibly [1]. Depending on conditions, the light-induced reactions of azabenzenes (pyridine, pyridazine, and pyrazine) can also give rise to various product mixtures and involve Dewar benzene-type primary intermediates [1,2,3].

Pure pyridine is a highly hygroscopic colorless photosensitive liquid. This aromatic heterocyclic compound reversibly converts into 5-amino-2,4-pentadienal (**1**) [4,5,6], also via Dewar pyridine [7], in the presence of water upon irradiation at 254 nm. The photoinduced formation of substituted pyridine derivatives via pyridinyl radicals is an alternative mechanism observed in non-polar aliphatic solvents such as cyclohexane [8]. The reversible ring-opening of pyridine in water was proposed as an effective undergraduate experiment [9]. According to the authors, the aldehyde enamine derived from glutaconic aldehyde is unstable and reverts slowly, at room temperature in the dark, to pyridine. Interestingly, the process is rendered irreversible in the presence of strong basic or acidic media, wherein ammonia and glutaconic acid are produced. A pure sample of 5-amino-2,4-pentadienal was isolated as yellow crystals, which rapidly turned brownish at room temperature and upon exposure to air [10]. The (almost) reversible formation of 5-amino-2,4-pentadienal upon 10 min irradiation at 250 nm of a poly(4-vinyl pyridine)/pyridine/water system was proposed [11] to be responsible for the appearance of a multi-band emission spectrum extending above 600 nm.

Recently, we suggested that the appearance of the multi-band emission spectrum of pyridine, irradiated at 254 or 312 nm, cannot be explained by the formation of 5-amino-2,4-pentadienal alone and suggested that this aminoaldehyde undergoes oligomerization [12]. Gaining deeper insight into the photochemical processes within pyridine and its derivatives is important, in particular, since polyvinyl pyridines are known to exhibit potential for several industrial applications. For instance, poly(4-vinyl pyridine) (P4VP) and its photochemical behavior attracted researchers as a promising material for photolithography [13] and references therein. Cross-linking by UV irradiation at 254 nm, which is ascribed to recombination of the photogenerated radical species, was used for the preparation of negative tone photoresists. Thoroughly dried P4VP dissolved in dry pyridine, taken in the equimolar ratio to the pyridine repeat units of the polymer, produces stable solid photo- and electrosensitive gels P4VP/Py [14]. The physical aggregation of polymer chains was shown to occur due to the presence of the hydrogen bond network, and these gels exhibit not only high ionic conductivity (polyelectrolyte behavior), but also enhanced photoinduced electron mobility [15,16]. The P4VP/Py gel was shown to exhibit unusual emission with the maxima at 477, 527, and 584 nm, explained by photoinduced proton transfer [17], which resembled the emission from the irradiated pyridine [12]. Here, we report on our investigation of the photochemical behavior of low-molecular-weight poly(4-vinyl pyridine)/pyridine (P4VP/Py) gels.

## 2. Results and Discussion

For our initial experiments, we used the commercially available P4VP polymer with a molecular weight of 50,000 g/mol. To increase the solubility of photoproducts and enable ^1^H-NMR monitoring, we prepared a low-molecular-weight polymer (MW about 2000 g/mol) (see Supplementary Materials for the details). Freshly distilled pyridine and thoroughly dried polymer samples were used, and although this treatment is insufficient to completely remove water, we noticed that the photoinduced reactions occur more rapidly.

**Scheme 1:**
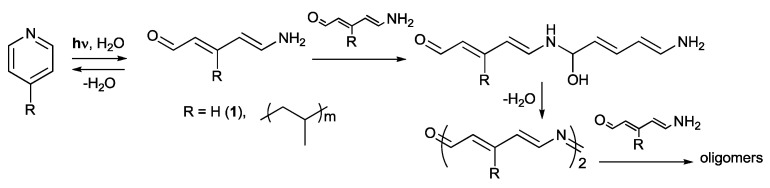
Photoinduced pyridine ring-opening and subsequent polycondensation.

The efficient photoinduced pyridine ring-opening, recyclization, and polycondensation require the presence of only a catalytic amount of water, as shown in Scheme 1.

**Figure 1:**
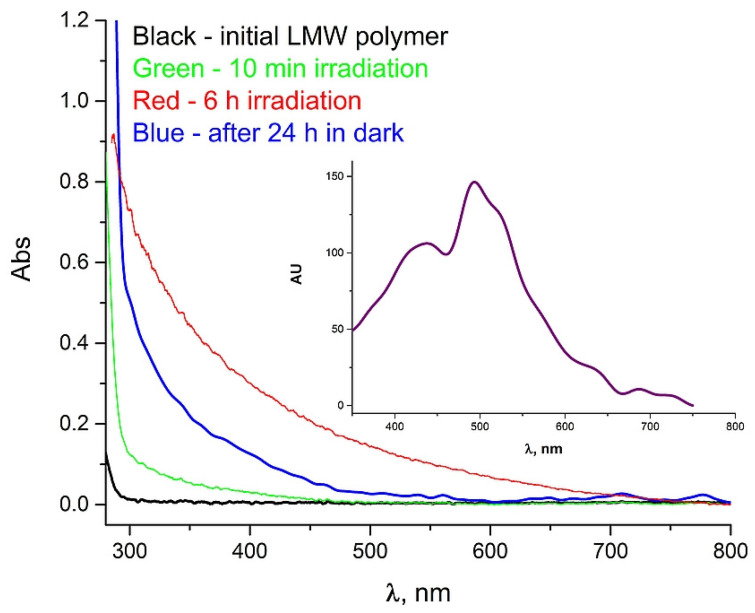
UV-Vis spectra of low-MW P4VP/Py gel samples in methylene chloride before and after irradiation at 312 nm as well as after holding the 6 h irradiated sample in the dark for 24 h. Inset: fluorescence spectrum of a thin film of a sample irradiated for 6 h (excitation at 350 nm).

The new P4VP/Py gel composition was prepared from the low-MW polymer as thick, viscous oil. Irradiation of a thin-layer sample of this gel on a quartz plate gave rise to the appearance of yellow to yellow–brownish coloration. The products were partially soluble in methylene chloride, and the respective changes in the UV-Vis spectra observed upon irradiation in methylene chloride solution are shown in Figure 1. Although absorption of a dilute solution of P4VP/Py gel is very weak at 312 nm, after 6 h of irradiation, the absorption of the products extends to 750 nm, and after 24 h in the dark, the methylene chloride solution became slightly turbid, indicative of a thermal-induced increase in cross-linking density. No more changes in absorption were observed after prolonged storage in the dark. The fluorescence spectrum of a thin layer of the gel sample irradiated for 6 h shown in the inset of Figure 1 covers the whole visible range and resembles the fluorescence spectrum of the irradiated pyridine samples [12]. The intensity of several peaks and shoulders observed in the fluorescence spectrum of irradiated pyridine depend on the excitation wavelength, and each of them belongs to a different soluble oligomer chain length (Supplementary Materials, Figure S2). We did not notice any difference between the samples irradiated at 254 and 312 nm.

**Figure 2:**
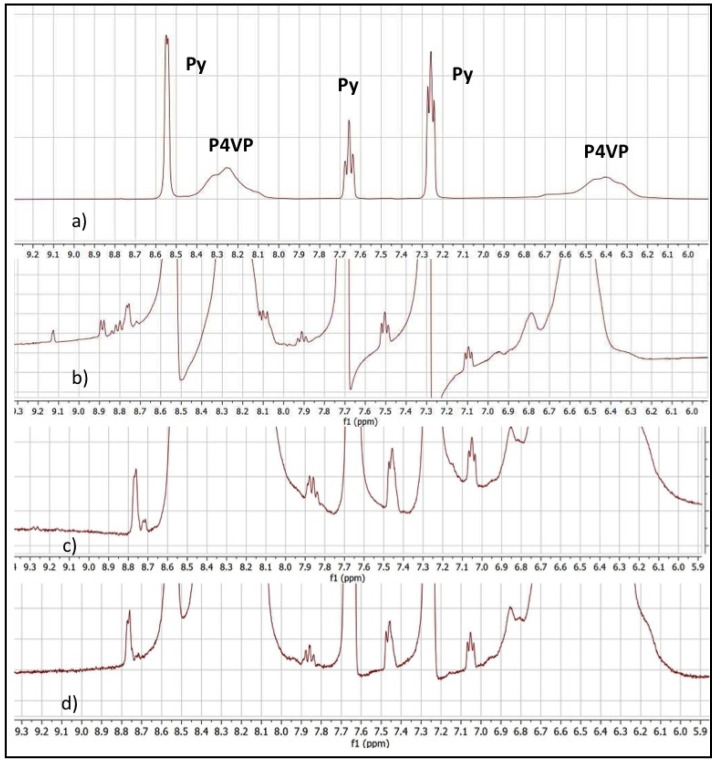
(**a**) ^1^H NMR spectrum of P4VP/Py gel before irradiation; (**b**) after 1 h irradiation of P4VP/Py gel at 312 nm; (**c**) after 6 h irradiation at 312 nm; (**d**) after 6 h irradiation of P4VP/Py gel and subsequent 24 h storage in the dark.

The higher solubility of the gel made from pyridine, low-MW P4VP, and its photoproducts in methylene chloride allowed monitoring of the process using the ^1^H-NMR technique (Figure 2). The downfield part of the gel spectrum before irradiation exhibited two broad signals at about 8.25 and 6.35 ppm, characteristic of pyridine pendant group protons (Figure 2a). Irradiation of the neat gel at 312 nm for one hour was followed by dissolution of the sample in CD_2_Cl_2_ and immediate ^1^H-NMR spectrum recording (Figure 2b). The presence of a mixture of the pyridine-ring-opened products can be recognized by a doublet signal at 9.12 ppm, which is assigned to the proton of the terminal CH=O group present in 5-amino-2,4-pentadienal and corresponding self-condensation oligomers, as well as a doublet at 8.77 ppm, which can be assigned to the proton of the HC=N- group present in the oligomers [18]. The spectrum of the solvent-free P4VP/Py gel sample after irradiation for 6 h is shown in Figure 2c. The increased amount of longer oligomers is manifested by the increased intensity of the polyene =C-H group signals and the diminished intensity of the downfield doublet of the O=C-H group. This doublet signal, at about 9.3 ppm, is still discernible after 6 h of irradiation (Figure 2c), but disappears completely after storing the irradiated sample for 24 h in the dark (Figure 2d). At the same time, the intensity of the signal at 8.77 ppm, corresponding to the HC=N group, increases in the dark as compared to the signal of pyridine (Figure 2c,d). Notably, the positions and signal-splitting patterns (doublets and triplets, the latter being actually doublets of doublets) are similar to those previously observed for 5-amino-2,4-pentadienal [10] and a series of azapolyenealdehydes in DMSO*_d6_* [18].

**Chart 1:**
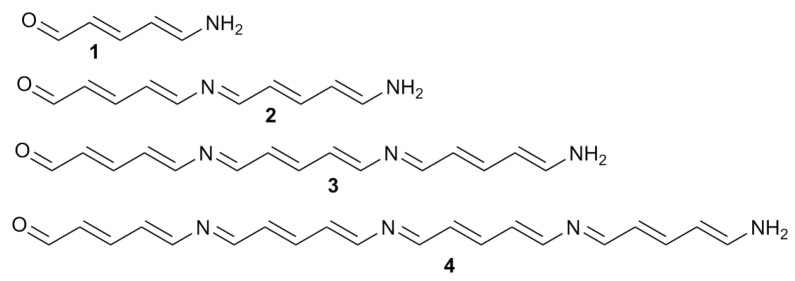
1,6-Polyazaacetylene monomer (**1**) and respective oligomers (**2**–**4**): *all-trans* model structures.

In order to gain a deeper insight into the electronic structures of the 5-amino-2,4-pentadienal polycondensation products, which are actually oligomers of hitherto unknown polyazaacetylenes, we undertook quantum mechanical calculations [19] on a series of oligomers of **1** (Chart 1) (see Supplementary Materials for the computational details).

**Figure 3:**
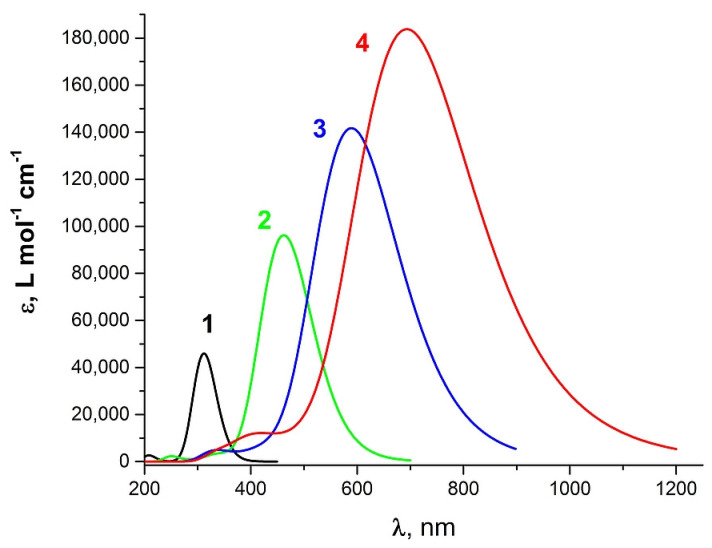
Calculated (B3LYP/aug-cc-pVDZ//B3LYP/aug-cc-pVDZ model chemistry) absorption spectra of molecules **1**–**4** (Chart 1) in dichloromethane; TD-SCF was solved for 10 states.

Even taking into account that the structures in the crowded pendant-conjugated oligomers can be far from the ideal *all-trans* conformation, and that chain length growth can be limited by the solubility of longer oligomers, the presence of trimer **3** can already provide absorption in the whole visible range (Figure 3).

We also calculated the vertical ionization potentials (IP) and electron affinities (EA) of the model compounds characterizing their electron-donating and -accepting properties (Table 1). Whereas monomer **1** is a moderate donor comparable to anthracene (experimental IP = 7.40 eV), the IP of dimer **2** is close to that of pentacene (IP = 6.64 eV), and those of **3** and **4** are very strong electron donors which can be compared to tetrathiafulvalenes (IP = 6.38 − 6.92 eV), well-known components of organic metals [20]. According to calculations, compounds **1** and **2** are weak acceptors, but the EA of compound **3** compares to *p*-benzoquinone (EA = 1.85 eV), and derivative **4** to tetracyano-*p*-benzoquinone (2.45 eV) (for an experimental IP and EA data compilation see [20]).

**Scheme 2:**
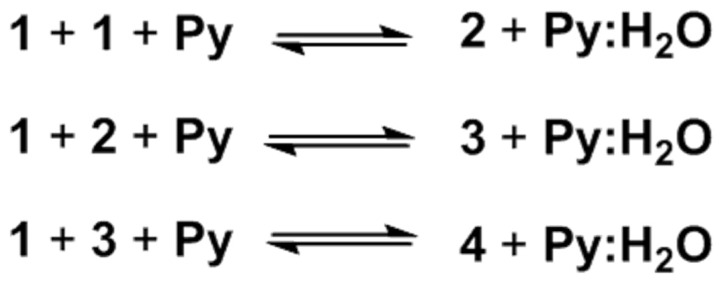
Stepwise polycondensation reactions of **1**.

**Figure 4:**
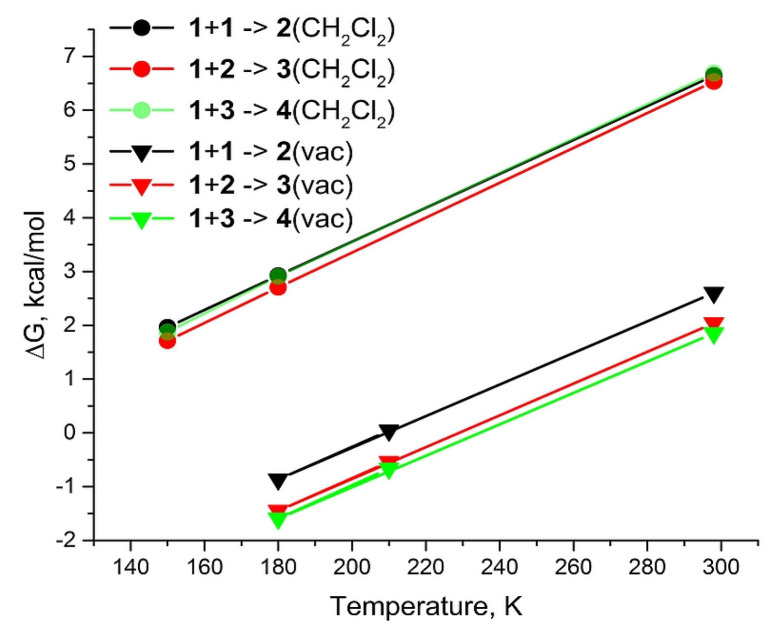
Calculated ΔG of the proposed polycondensation reactions in dichloromethane and in vacuum.

A possible polycondensation process is presented in Scheme 2. A molecule of pyridine was taken into account owing to its hygroscopicity; it forms strong hydrogen bonds with at least one molecule of water (binding enthalpies of 4.5−5.9 kcal/mol according to [21], and our present calculations afforded 4.8 kcal/mol), thus serving as a water-binding component. We find that in both the dichloromethane solution and in a vacuum, the ΔH values of the reactions are always negative and amount to −2.4 to −2.9 and −5.9 to −6.5 kcal/mol at 298 K in the solution and vacuum, respectively. The calculated ΔG values are presented in Figure 4. The Gibbs free energies became negative below about 80 K, as calculated in methylene chloride, and about 210 K in the vacuum. These values are typical for polycondensation reactions [22,23]. A large difference in the temperatures at which the polycondensation becomes thermodynamically favorable (a polar solvent vs. vacuum) is related to the electron-donating–electron-accepting (D–A) conjugated structure of the polycondensing molecules: the degree of charge transfer from D to A increases in polar media, giving rise to stabilization of the monomer and decrease in ΔH of polycondensation reactions. Moreover, the presence of regions of local order within the gels can render the polycondensation process enthalpy-driven rather than entropy-driven.

**Figure 5:**
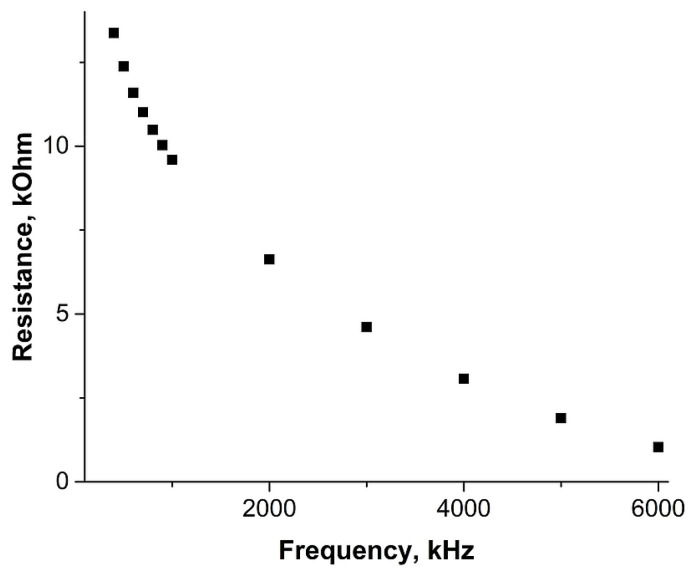
Resistance dependence on the AC voltage frequency of the low-MW gel. Voltage amplitude 35 mV, thickness of the sample 230 μm, cross-section 5 × 5 mm.

Samples of gels prepared from low- and high-MW polymers exhibit similar electrochemical behavior. Both gels possess polyelectrolyte properties, and their conductivity originates from ion mobility. The resistance of the gels depends on the voltage frequency, as shown in Figure 5.

**Figure 6:**
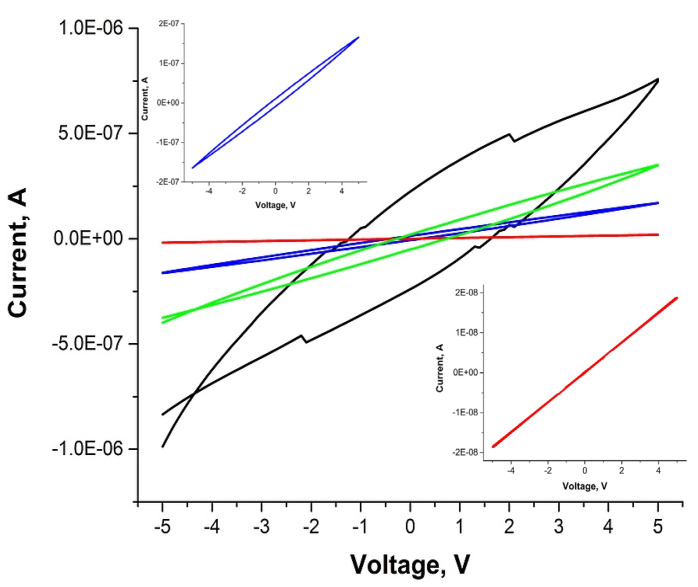
Current–voltage curves of low-MW P4VP/Py gel sample (thickness 230 mm, cross-section 5 × 5 mm) before irradiation (black curve); after irradiation at 254 nm for one hour and 10 h holding in the dark (green curve); after additional one-hour irradiation and holding the sample in the dark for 10 h (blue curve and **upper** inset); after the third one-hour irradiation and holding the sample in the dark for 10 h (red curve and **lower** inset). The sweep rate was 1.7 V/s.

The electrical properties of the gels were recorded using a home-made setup (Supplementary Materials, Figure S3). The conductivity of the gels undergoes remarkable changes upon irradiation, as shown in Figure 6. Thus, after one-hour irradiation at 254 nm, and holding the low-MW P4VP/Py gel sample for 10 h in the dark, the conductivity and hysteresis typical of ionic conductivity diminished. After repeating the above irradiation/holding in the dark treatment two more times, we found that the dependence of the current on the applied voltage becomes linear, following Ohm’s law (Figure 6). This is a direct indication of the electronic conductivity mechanism.

Similar behavior was observed in the voltammograms of the high-MW P4VP/Py gel samples. The conversion of ionic conductivity into electronic conductivity is irreversible, as the prolonged holding of the irradiated samples in the dark does not lead to even partial re-appearance of the hysteresis. This also means that the regions of local order within the gels cannot be restored after irradiation. This phenomenon can be noticed in transmission electron microscopy (TEM) images of the samples before and after irradiation (Supplementary Materials, Figure S4).

## 3. Conclusions

We conclude that the color and electrical property changes of both pyridine and P4VP/Py gels occur as a result of the initial photoinduced pyridine ring-opening, which produces 5-amino-2,4-pentadienal (**1**) or/and the 5-amino-2,4-pentadienal moieties attached to the polyvinyl chain, and its subsequent polycondensation into the hitherto unknown oligomeric polyazaacetylenes. The oligomers can further cross-link the polymer chains. TD DFT calculations show that polyazaacetylenes possess both strong electron-donating and electron-accepting properties. The later attribute facilitates the occurrence of redox process events between the chains, or the reaction with oxygen and water to produce oxidized and reduced stable radical species. The consumption of the redox reactive polycondensation products shifts the equilibrium toward oligomer formation. The conjugated polycondensation cross-linked macromolecular system described herein serves as a ‘molecular wire’ converting the ionic conductivity of the P4VPy/Py gels into electronic conductors. The isolation and full characterization of the soluble oligomers produced by irradiation at low temperatures are under investigation in our laboratories.

## Figures and Tables

**Table 1:** Calculated vertical ionization potentials (IP) (eV) and electron affinities (EA) (eV) of model structures **1**–**4** (Chart 1) in vacuum (B3LYP/aug-cc-pVDZ//B3LYP/aug-cc-pVDZ model chemistry).

Structure	IP (eV)	EA (eV)
**1**	7.39	0.26
**2**	6.60	1.21
**3**	6.23	1.84
**4**	6.01	2.23

## Data Availability

The data presented in this study are available in Supplementary Materials.

## Supplementary Materials

The following are available online. Experimental and computational details: synthetic procedure for low-molecular-weight poly(4-vinylpyridine) and ^1^H-NMR spectrum (Figure S1); fluorescence spectra of irradiated pyridine at different excitation wavelengths (Figure S2); gel preparation, spectrometer details, calculation details, and the full reference to Gaussian 16 software; conductivity measurements setup (Figure S3); and TEM images of the low-MW samples of gel before and after irradiation (Figure S4).

## Sample Availability

Samples of the compounds are not available from the authors.
