# Supplementary material for: Light-Induced Reactions within Poly(4-vinyl pyridine)/Pyridine Gels: The 1,6-Polyazaacetylene Oligomers Formation
Source: Molecules. 2021 Nov 17;26(22):6925. doi: 10.3390/molecules26226925 (PMC8621047; doi:10.3390/molecules26226925)
Supplement: Supplementary file 1 [file molecules-26-06925-s001.zip › molecules-1457555-supplementary.pdf]

Light-induced reactions within the poly(4-vinyl pyridine)/pyridine gels: the 1,6-polyazaacetylene oligomers formation

Evgenia Vaganova,[a] Dror Eliaz,[a] Ulyana Shimanovich,*[a] Gregory Leitus,[b] Emad Aqad,*[c] Vladimir Lokshin,[d] Vladimir Khodorkovsky.*[d]

[a] Department of Molecular Chemistry and Materials Science, Weizmann Institute of Science, 7610001, Rehovot, Israel
[b] Chemical Research Support Department, Weizmann Institute of Science, 7610001, Rehovot, Israel
[c] DuPont Electronics & Industrial, Marlborough, Massachusetts, 01762 USA
[d] Aix Marseille Univ, CNRS UMR 7325, Centre interdisciplinaire de Nanoscience de Marseille (CINaM)Campus de Luminy, 13288 Marseille cedex 09, France; khodor@cinam.univ-mrs.fr

Experimental and computational details.

1. Synthesis of low molecular weight Poly(4-Vinylpyridine) (P4VP): Solution free radical homopolymerization of 4-vinylpyridine was performed in N,N-dimethylformamide (DMF) at 80 °C. Freshly distilled 4-vinylpyridine (25.0 g, 238.0 mmol), dry DMF (29.5 mL) and Dimethyl 2,2'-azobis(2-methylpropionate) (4.38 mg, 19.0 mmol) were charged into a 100 mL reaction flask. The mixture was purged with nitrogen for 0.5 h. The flask was immersed into an oil bath set at 80 °C and the reaction mixture was stirred under nitrogen for 3 h. The reaction mixture was cooled to room temperature and poured slowly into methyl *t*-butyl ether (500 mL). The crude polymer was collected by filtration and dried under vacuum at 50 °C. The crude polymer was subject to second precipitation from isopropanol (50 mL) into methyl *t*-butyl ether (500 mL) followed by filtration and drying in vacuo at 50 °C for 2 days. Yield: 14.5 g (58.0 %). The number average molecular weight (Mw) determined by ^{1}H NMR end-group analysis was 1976 (an average of 18.8 repeat units).

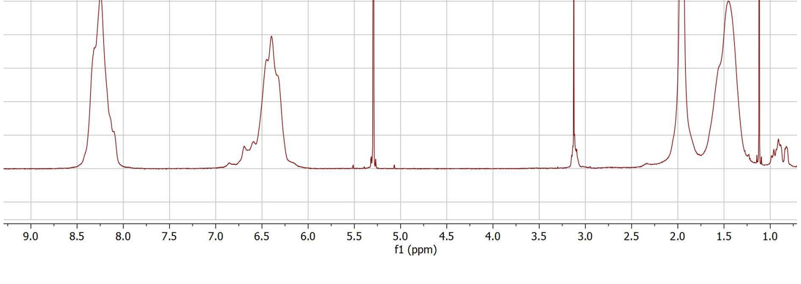

Figure S1. ^{1}H-NMR spectrum of P4VP in dichloromethane$_{d2}$.

2. Gel preparation. Gels were prepared using a standard procedure. Both P4VP with molecular weight 50 000 g/mol (Polyscience, Inc.) and molecular weight about 2 000 g/mol were carefully dried in a vacuum oven (10^{-3} Torr) at room temperature for a few days prior to use. Anhydrous pyridine (<0.003% water) from Aldrich was used. The polymer gels were prepared by mixing P4VP with pyridine in the ratio between free solvent and side-chain Py groups 1:1 and stored in the dark for about a week. Irradiation of the samples, UV-Vis spectra and conductivity measurements were done using quartz and quartz/ITO plates as shown in Figure S2.
3. The UV–Vis absorption spectra were recorded with Ocean Optics USB 4000 and JASCO V-660 spectrophotometers. ^{1}H-NMR spectra were recorded with a JEOL JNM-ECS400 spectrometer at 399.78 MHz. Chemical shifts are given in ppm downfield from tetramethylsilane.
4. Samples irradiation. A UV source (WFH-204B Ultraviolet Analyzer Lamp (www.cit17.com)), consisting of a mercury vapor lamp, with an emission peak at 254 nm operating at a nominal 4W. The intensity of the UV-light in the place of sample location was measured by Power meter (OPHIR) with Si/SiO2 wide range detector. The intensity of 60 μW/cm2 was measured. UV Transilluminator UST-2OM-8R (Biostep), 312 nm was used for irradiation at 312 nm.
5. Quantum-mechanical calculations were done using Gaussian 16, Rev. A.03 software [S1], B3LYP/aug-cc-pVDZ//B3LYP/aug-cc-pVDZ model chemistry. Geometry optimizations of the model structures, pyridine, and water were done using tight conversion criteria and Gaussian 16 defaults (in particular, integral=ultrafine). Harmonic frequency calculations at the same level verified achieving the energy minima (zero imaginary frequencies) and provided the estimates of the free energies G. All calculations in dichloromethane were carried out using the default self-consistent reaction field (SCRF) model. The absorption spectra were calculated using TD-SCF solved for 10 states. Vertical ionization potentials

(IP) and electron affinities (EA) were calculated according to their definitions as IP = E(M+.) – E(M) and EA = E(M) – E(M-.) (eV), where M is any of compounds **1 – 4**. The energy of the hydrogen bond between pyridine and water was calculated as the difference between the energies of these two molecules and the energy of a geometry optimized complex of both with the water molecule positioned in proximity to the nitrogen atom of pyridine and subsequent harmonic frequency calculations.

6. Conductivity measurements. I-V measurements were done in a two-electrode configuration (Figure S2). Copper wires were connected to the ITO conductive surfaces. Measurements were done when the sample was placed in Janis ST-500-2 probe station. DC and AC electric measurements were done using Keithley-4200A SCS system controlling Clarius software. DC I/V measurements were performed by means of 4200-SMU (source-measure unit) in the interval from -5 V to +5 V. AC measurements of impedance were carried out using 4210-CVU (capacity-voltage unit) applying RMS voltage 35 mV in the frequency range 1 kHz - 10 MHz. Different sweeping rates were applied. To confirm the reproducibility of the results, the experiments were repeated several (up to 10) times.

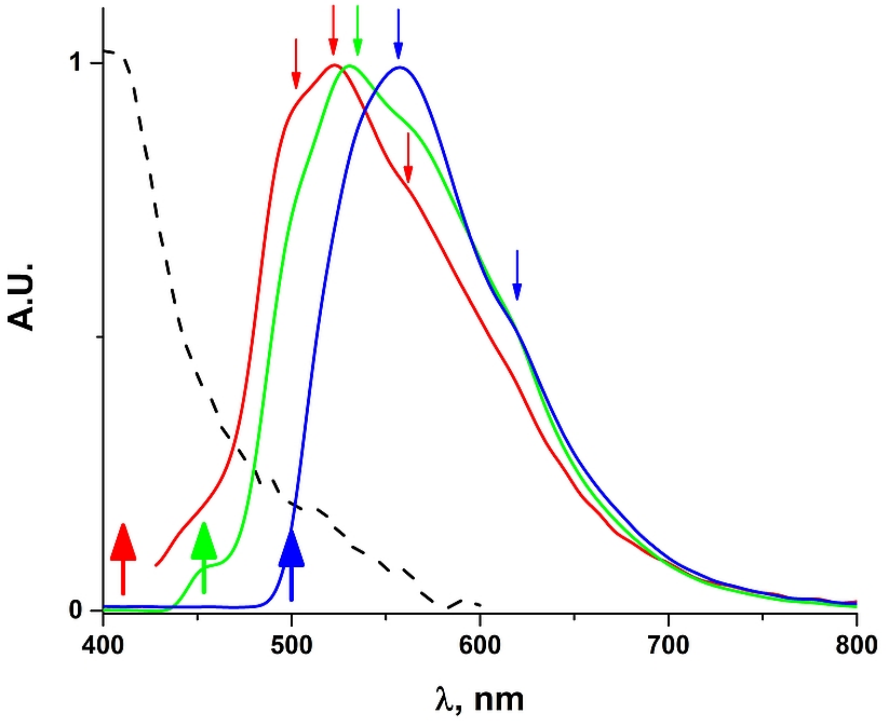

Figure S2. Pyridine absorption (dashed curve) and fluorescence after 10 min irradiation at 312 nm. Red: excitation at 410 nm, green: excitation at 450 nm, blue: excitation at 505 nm.

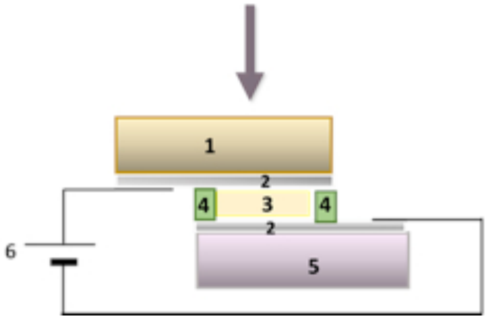

Figure S3. 1, 5 - quartz plates; 2 - conductive ITO layers (not used for spectra recording); 3 - gel; 4 - designed glass spacer, thickness 230 μm, cross-section 5 x 5 mm; 6 – bias supplied voltage.

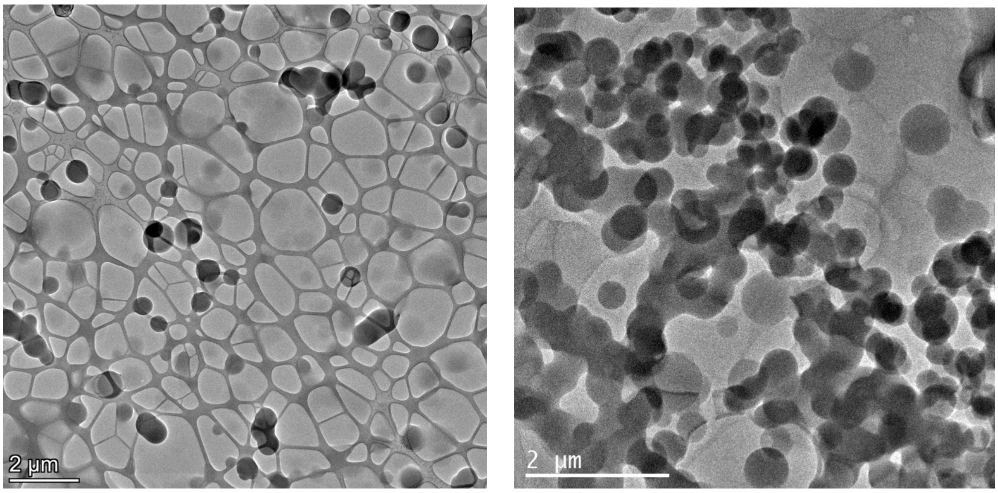

a) b)

Figure S4. TEM images of the low Mw samples of gel. a) before and b) after irradiation.

Full reference [19]:

M. J. Frisch, G. W. Trucks, H. B. Schlegel, G. E. Scuseria, M. A. Robb, J. R. Cheeseman, G. Scalmani, V. Barone, G. A. Petersson, H. Nakatsuji, X. Li, M. Caricato, A. V. Marenich, J. Bloino, B. G. Janesko, R. Gomperts, B. Mennucci, H. P. Hratchian, J. V. Ortiz, A. F. Izmaylov, J. L. Sonnenberg, D. Williams-Young, F. Ding, F. Lipparini, F. Egidi, J. Goings, B. Peng, A. Petrone, T. Henderson, D. Ranasinghe, V. G. Zakrzewski, J. Gao, N. Rega, G. Zheng, W. Liang, M. Hada, M. Ehara, K. Toyota, R. Fukuda, J. Hasegawa, M. Ishida, T. Nakajima, Y. Honda, O. Kitao, H. Nakai, T. Vreven, K. Throssell, J. A. Montgomery Jr., J. E. Peralta, F. Ogliaro, M. J. Bearpark, J. J. Heyd, E. N. Brothers, K. N. Kudin, V. N. Staroverov, T. A. Keith, R. Kobayashi, J. Normand, K. Raghavachari, A. P. Rendell, J. C. Burant, S. S. Iyengar, J. Tomasi, M. Cossi, J. M. Millam, M. Klene, C. Adamo, R. Cammi, J. W. Ochterski, R. L. Martin, K. Morokuma, O. Farkas, J. B. Foresman and D. J. Fox, Gaussian 16, Revision A.03, Gaussian, Inc., Wallingford CT, 2016.
